# Citronellol Induces Apoptosis via Differential Regulation of Caspase‐3, NF‐κB, and JAK2 Signaling Pathways in Glioblastoma Cell Line

**DOI:** 10.1002/fsn3.4678

**Published:** 2025-01-06

**Authors:** Muhammad Nasir Hayat Malik, Sufyan Ali, Amir Ali, Abdullah R. Alanzi, Muhammad Atif, Hattan A. Alharbi, Bowen Wang, Moosa Raza, Tahir Maqbool, Irfan Anjum, Shah Jahan, Saud O. Alshammari, Gideon F. B. Solre

**Affiliations:** ^1^ Faculty of Pharmacy The University of Lahore Lahore Pakistan; ^2^ Department of Pharmacognosy, College of Pharmacy King Saud University Riyadh Saudi Arabia; ^3^ College of Chinese Medicine Hubei University of Chinese Medicine Wuhan Hubei China; ^4^ Institute of Molecular Biology and Biotechnology (IMBB) The University of Lahore Lahore Pakistan; ^5^ Shifa College of Pharmaceutical Sciences Shifa Tameer‐e‐Millat University Islamabad Pakistan; ^6^ Department of Immunology University of Health Sciences Lahore Pakistan; ^7^ Department of Pharmacognosy and Alternative Medicine, College of Pharmacy Northern Border University Rafha Saudi Arabia; ^8^ Department of Chemistry, Thomas J. R. Faulkner College of Science and Technology University of Liberia Monrovia Montserrado County Liberia

**Keywords:** anticancer, caspase‐3, citronellol, glioblastoma, NF‐κB

## Abstract

Citronellol (CT) is a naturally occurring lipophilic monoterpenoid which has shown anticancer effects in numerous cancerous cell lines. This study was, therefore, designed to examine CT's potential as an anticancer agent against glioblastoma (GBM). Network pharmacology analysis was employed to identify potential anticancer targets of CT. A comprehensive data mining was carried out to assess CT and GBM‐associated target genes. Protein–protein interaction network was constructed to identify hub genes and later GO and KEGG enrichment analysis was performed to elucidate the possible mechanism. Human glioblastoma cell line “SF767” was used to confirm in silico findings. MTT, crystal violet, and trypan blue assays were performed to assess the cytotoxic effects of various concentrations of CT. Subsequently, ELISA and qPCR were performed to analyze the effects of CT on proapoptotic and inflammatory mediators. In silico findings indicated that CT differentially regulated proapoptotic and inflammatory pathways by activating caspase‐3 and 8 and inhibiting nuclear factor‐kappa B (NF‐κB), tumor necrosis factor‐α, Janus kinase 2 (JAK2). Molecular docking also demonstrated strong binding affinities of CT with the above‐mentioned mediators when compared to 5‐fluorouracil or temozolomide. In SF767 cell line, CT displayed dose‐dependent cytotoxic and antioxidant effects, and upregulation of annexin‐V, caspase‐3, and 8 along with downregulation of inflammatory modulators. In a nutshell, it can be concluded from these findings that CT possesses robust anticancer activity which is mediated via differential regulation of caspase‐3, JAK2, and NF‐κB pathways.

## Graphical abstract

Citronellol has emerged as a promising anticancer agent against glioblastoma (GBM). Network pharmacology analysis and experimentation on glioblastoma cell line have confirmed the beneficial effects of citronellol in GBM.

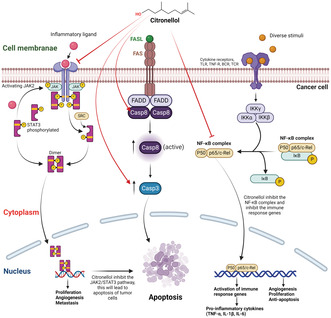

## 1 Introduction

Cancer is a disease characterized by uncontrollable division of cells, resulting in excessive morbidity and mortality rates (Al‐Ostoot, Salah, and Khanum 2024). Glioblastoma (GBM) is a malignant tumor originating from the rapidly proliferating cells within the brain or spinal cord (Roda et al. 2024). These tumors develop due to cancerous growth of glial cells and can infiltrate neighboring tissues (Roda et al. 2024). Macroscopically, GBM often shows a variety of features, including multifocal hemorrhage, necrosis, and cystic and gelatinous areas, resulting in regions of necrosis and varied levels of blood supply (Hanif et al. 2017).

Gliomas are the predominant forms of primary intracranial tumors accounting for about 80% in adolescents (Wagner et al. 2024). GBM is the deadliest glioma generally classified as WHO grade IV having an average survival time of 15 months (Grochans et al. 2022; Liang et al. 2020). The incidence rate of gliomas varies from 3.19 to 4.17 per 100,000 persons‐years (Grochans et al. 2022; Segura et al. 2023). Pediatric GBM accounts for 3%–15% out of all brain tumors reported in children (0–18 years) with an incidence rate of 0.85 per 100,000 persons‐years (Ostrom et al. 2022; Singla et al. 2021).

To date, there is no permanent cure for GBM (Rajaratnam et al. 2020) and the treatment options for GBM are complex which include tumor removal, radiation therapy followed by chemotherapy with temozolomide (TMZ) (Yalamarty et al. 2023). Numerous anticancer agents have been investigated in an attempt to enhance the survival of GBM patients (Hanif et al. 2017). Alkylating medications, such as lomustine (CCNU), carmustine (BCNU), and TMZ, exhibit therapeutic potential in the treatment of GBM. However, BCNU and CCNU exhibit harsh cytotoxicity, leading to early development of resistance and various side effects. The best adjuvant therapeutics after surgical resection are radiotherapy and TMZ, but these can prolong the lifespan for a few months only (Tzitiridou et al. 2024). Studies have indicated that TMZ resistance has emerged due to an upregulation in O6‐methylguanine‐DNA methyltransferase (MGMT) activity. GBM tumors usually develop resistance against TMZ due to overexpression of MGMT and recurrences have been reported in several clinical cases (Singh 2021). Although much progress has been made in terms of treatment methods still the future looks bleak for gliomas due to their extremely aggressive, vascularized, and infiltrating pathological features, thereby, necessitating new treatments (Su et al. 2024). Therefore, there is a need to quest for an alternative agent which can efficiently treat and manage GBM.

Natural compounds have gained much attention over the years in pharmaceutical research owing to their potent pharmacological actions and better safety profiles (Chopra and Dhingra 2021). In recent days, citrus consumption has increased worldwide as it contains essential oils which are known to reduce the risks of various chronic diseases including a wide variety of cancers (Abakpa and Adenaike 2021; Li et al. 2010). Citronellol (CT), also known as 3, 7‐dimethyl‐6‐octen‐1‐ol, is a naturally occurring acyclic monoterpenoid (Gandhi et al. 2024), which is found in significant amounts in citrus fruits and over 70 essential oils, with Bulgarian rose oil and geranium oil being the primary sources (Rajendran, Pachaiappan, and Thangarasu 2021). Studies have shown that CT possesses potent antioxidant, antidiabetic, anticancer, cardioprotective, and anti‐inflammatory properties (Fadwa et al. 2022; Rajendran, Pachaiappan, and Thangarasu 2021). In vitro investigations indicate that CT can suppress the proliferation of human lung cancer, hepatocellular carcinoma, hypopharyngeal carcinoma, and breast cancer cells (Fatima and Luqman 2021; Rajendran, Pachaiappan, and Thangarasu 2021; Widiyarti, Handayani, and Hanafi 2018; Yu et al. 2019). In an animal model of Parkinson's disease, CT penetrated the blood–brain barrier and proved to be effective in protecting dopaminergic neurons due to its antioxidant and anti‐inflammatory properties (Jayaraj et al. 2022). Based on its reported anticancer and anti‐inflammatory potentials, it was assumed that CT might be effective in treating the progression of GBM. Keeping in view the diverse pharmacological profile of CT, this study was conducted to mechanistically elucidate the anticancer potential of CT against GBM cell line “SF767.”

## 2 Materials and Methods

### 2.1 Network Pharmacology Analysis

#### 2.1.1 CT‐Associated Target Prediction

The GeneCards collects gene‐related information from 150 web sources covering genomic, transcriptomic, proteomic, genetic, clinical, and functional aspects of human genes. The similarity ensemble approach (SEA) is an effective database for drug development that predicts chemical similarities and side effects. SEA classifies proteins based on the chemical similarity of their ligands. SEA can identify previously unknown associations between ligand and receptors by categorizing receptors based on their chemical similarity (Wang et al. 2016). WAY2DRUG (DIGEP‐Pred 2.0) is a web service that predicts drug‐induced changes in gene expression profiles using the structural formulas of drug‐like compounds. This comprises gene expression changes from the Comparative Toxicogenomics Database (CTD) at both the mRNA and protein levels, as well as Connectivity Map (cMAP) build02 (Lagunin et al. 2013). Swiss Target Prediction utilizes a large library of 370,000 active chemicals to suggest potential targets for over 3000 proteins from three species (Gfeller et al. 2014). The CT‐related target database was developed by integrating the retrieved targets and removing repetitive targets.

#### 2.1.2 GBM‐Associated Target Prediction

The GBM‐associated targets were retrieved from GeneCards, DisGeNET and Open Targets Platform database and were screened based on gene–disease association scores. The disease genes based on database were selected on the basis of scoring criteria and only *Homo sapiens* proteins were selected. DisGeNET is a comprehensive discovery platform that contains over 380,000 associated between more than 16,000 genes and 13,000 diseases making it one of the largest archives (Piñero et al. 2015). The Open Targets Platform (OTP) builds and scores target–disease associations which aid in therapeutic target identifications. It integrates publicly available datasets, including data generated by the Open Targets consortium (Ochoa et al. 2021).

### 2.2 Construction of Protein–Protein Interaction (PPI) Network

We used Cytoscape 3.10.2 to create and analyze the networks for screened molecular targets. This open software platform allows for network design, visualization, analysis, identifying target proteins, and their relationships with compounds as well as to view the pathways and diseases involved (Shannon et al. 2003). We used the STRING database to predict PPIs for hub genes in *H. sapiens* , focusing on those with a binding score of 0.9 or higher (Mering et al. 2003). The targets were reintroduced in Cytoscape to visualize the PPIs network. The most linked hubs or nodes in the network were identified and ranked by using betweenness centrality, a crucial topological metric.

### 2.3 Gene Ontology and Functional Enrichment Analysis

Gene ontology (GO) enrichment analysis was performed using the DAVID database, which includes biological processes (BP), cellular components (CC), molecular functions (MF), and Kyoto Encyclopedia of Genes and Genomes (KEGG) pathways (Consortium 2006; Dennis Jr. et al. 2003). We used the SRplot platform to visualize our findings in bubble charts and bar chart format, demonstrating its usefulness as an online data analysis tool.

### 2.4 Molecular Docking

#### 2.4.1 Structures of Target Proteins

The 3D structures of human caspase‐8 (PDB ID: 3KJQ) at a resolution of 1.80 Å, human caspase‐3 (PDB ID: 1NME) at a resolution of 1.60 Å, human IKB‐α/NF‐KB1 complex (PDB ID: 1IKN) at a resolution of 2.30 Å, human JAK2 (PDB ID: 3JY9) at a resolution of 2.10 Å, and human TNF‐α (PDB ID: 2AZ5) at a resolution of 2.10 Å were retrieved from Research Collaboratory for Structural Bioinformatics (RCSB) in Protein Data Bank (PDB) format (Erlanson et al. 2003; He et al. 2005; Huxford et al. 1998; Wang et al. 2009; Wang et al. 2010). The structures of the different proteins were preprocessed by the MOE Protonate‐3D module to ensure their docking analysis, including the removal of any cocrystallized ligands, water molecules, and other heteroatoms as well as the addition of missing hydrogen atoms, and optimization of side‐chain orientations. The protein structures were then subjected to energy minimization using an AMBER99 force field to relax any steric clashes or structural imperfections (Figure S1).

#### 2.4.2 Ligand Preparation

The 3D structure of the ligand was taken from the PubChem database of the National Center for Biotechnology Information (NCBI) and was converted into pdb format using the BIOVIA discovery studio visualizer (Gao and Huang 2011). The ligand molecule was first preprocessed by MOE to remove counterions and salts to generate the appropriate protonation state. The ligand structure was then subjected to energy minimization using MMFF‐94x force field to obtain the most stable conformation (Figure S2).

#### 2.4.3 Active Binding Site Prediction of Target Proteins

The active binding sites of target proteins were predicted by the Computed Atlas of Surface Topography of Proteins (CASTP) (Tian et al. 2018). The binding site predicted for different proteins is listed in Table S1.

#### 2.4.4 Target Prediction

Screening for the optimal compounds against the proper molecular targets is a critical step in the drug discovery process (Nunez, Venhorst, and Kruse 2012). The structure of the ligand molecule was retrieved from ChemDraw Professional 16.0 in Simplified Molecular‐Input Line‐Entry System (SMILES) format and uploaded in the WAY2DRUG PASS prediction tool to determine the molecular targets of the ligand associated with GBM. This tool compares the input molecules with known compounds that have a certain potency using SAR analysis. A high prediction is obtained when there is a greater degree of structural similarity between the input chemical and known compounds (Filimonov et al. 2014). These identified targets were matched with genes associated with GBM, retrieved from DisGeNET database (Kaloni et al. 2020).

### 2.5 Methodology

Molecular docking and scoring calculations were performed by Molecular Operating Environment (MOE version 2019.0102). The docking study was conducted for ligands against different protein targets and then visualized using the BIOVIA discovery studio visualizer. First, the ligand database is made by MOE and converted into Microsoft Access database file (MDB) format. The input files were uploaded for docking analysis with 100 ligand conformations through MOE's default docking algorithm, that is, the Triangle Matcher for the method, London dG, and GBVI/WSA dG for scoring functions. After docking, the best‐docked result was selected by conformational visualization by BIOVIA.

### 2.6 SF767 Cell Lines

SF767 cell lines were provided by Cells and Tissue Culture laboratory of The University of Lahore. Cell lines were revived from cryovials that had been kept in liquid nitrogen and were used for culture whenever needed. The approval to conduct all studies was given by the “Institutional Research Ethics Committee” of the Department of Pharmacology, Faculty of Pharmacy, The University of Lahore, Lahore, Pakistan.

### 2.7 Cytotoxicity Evaluation of CT

Cell viability assay was conducted to find the optimal concentration of CT. Different dilutions of CT (1, 2, 5, and 10 mM) were formulated from 1 M stock solution. SF767 cells were seeded on a 96‐vial plate and incubated at 37°C overnight. Next day, medium from the wells was withdrawn and the cells were wiped with 1× PBS. Various concentrations of CT were introduced into the wells. Cell viability of treated cells was assessed by 3‐(4, 5‐dimethylthiazolyl‐2)‐2, 5‐diphenyltetrazolium bromide (MTT) assay according to the manufacturer's protocol (Maqbool et al. 2019).

### 2.8 Study Design

SF767 cells were divided into the following groups (*n* = 3 in each group):

1. Control: Complete DMEM medium
2. 5‐FU (200 μM): 200 μM of 5‐FU in complete DMEM medium
3. CT (1 mM): 1 mM of CT in complete DMEM medium
4. CT (2 mM): 2 mM of CT in complete DMEM medium
5. CT (5 mM): 5 mM of CT in complete DMEM medium
6. CT (10 mM): 10 mM of CT in complete DMEM medium

#### 2.8.1 Cell Viability Assay

In order to calculate the cell viability of treated SF767 cells, MTT and crystal violet (CV) assays were performed, in which different concentrations of above‐mentioned dilution were tested on SF767 cells cultured in 96‐well plates.

#### 2.8.2 MTT Assay

Treated cells were wiped with 1× PBS followed by 3–4 h of incubation with 100 μL of DMEM and 25 μL of MTT solution. Formazan crystals were solubilized with 10% sodium dodecyl sulfate (SDS) and absorbance was measured at 570 nm using microplate reader. Cell viability percentage was calculated from the mean absorbance values (Maqbool et al. 2019).

#### 2.8.3 CV Assay

Treated cells were rinsed with 1× PBS and treated with a mixture of 0.1% CV dye followed by incubation for 15 min at room temperature (RT). Wells were thoroughly washed with 1× PBS and dye was carefully disposed of to prevent cells from lifting out of the wells. The stain was later solubilized by adding 100 μL of 1% SDS to each well and being allowed for 5–10 min at RT. Finally, absorbance was measured at 595 nm using a microplate reader (Maqbool et al. 2019).

### 2.9 Dead Cell Detection

For dead cell detection, trypan blue assay was performed.

Trypan blue reagent has been used to distinguish between live and dead cells. Briefly, pretreated cells were washed three times with 1× PBS and subsequently stained with trypan blue (Cat. No. T6146). The blue‐stained cells were designated as dead, which were counted using compound microscope (Maqbool et al. 2019).

#### 2.9.1 ELISA Assay

ELISA was performed to measure caspase‐3 and annexin‐V using the cellular lysates. Assays were performed according to manufacturer's protocol (Lee et al. 2021).

#### 2.9.2 RT‐qPCR

Total RNA was extracted from SF767 cells using the standard TRIzol technique and WizScript cDNA Synthesis Kit (Wizbio solutions, New Mexico, USA; Cat. No. W2202) was used to reverse‐transcribe the RNA in accordance with the normal procedure. The ΔΔCT method was used to assess the transcript levels of several genes using “The Applied Biosystems StepOne Real‐Time PCR and Zokeyo 2xSYBR Green qPCR Mixture (Cat. No. HPR012‐01)” under the following conditions: Initial denaturation was carried out at 94°C for 2 min, followed by 40 cycles of denaturation at 94°C for 1 min, annealing at 60°C for 30 s, and elongation at 72°C for 15 s. As an internal standard, hypoxanthine–guanine phosphoribosyltransferase (HPRT) was used.

### 2.10 Statistical Analysis

Data from three biological replicates were represented as mean ± SD and were analyzed by one‐way ANOVA followed by Tukey's multiple comparison tests. All statistical analyses were carried out by using Graph Pad Prism 8.0 software. A probability of less than 0.05 was considered significant. The following abbreviations were used to demonstrate significance in the graph; ***≤ 0.001, **≤ 0.01, *≤ 0.05 (treated groups vs. control); $≤ 0.01, #≤ 0.001 (CT‐treated groups vs. 5‐FU‐treated group).

## 3 Results

### 3.1 Prediction of Pharmacological Spectrum

The pharmacological spectrum of CT was predicted by utilizing the WAY2DRUG (PASS) database. The “Probable activity” (Pa) and “probable inactivity” (Pi) were used to indicate the likely functions. Pa > Pi values were also taken into consideration during the biological spectrum interpretations. A total of five proteins were selected on the basis of their significantly down/upregulation (Table 1).

**TABLE 1:** Predicted pharmacological activity of CT.

Compound	Predicted activity
CT	Caspase‐3 stimulant
	Caspase‐8 stimulant
	Transcription factor NF‐κB inhibitor
	JAK2 expression inhibitor
	TNF expression inhibitor

### 3.2 Docking Analysis

**FIGURE 1:**
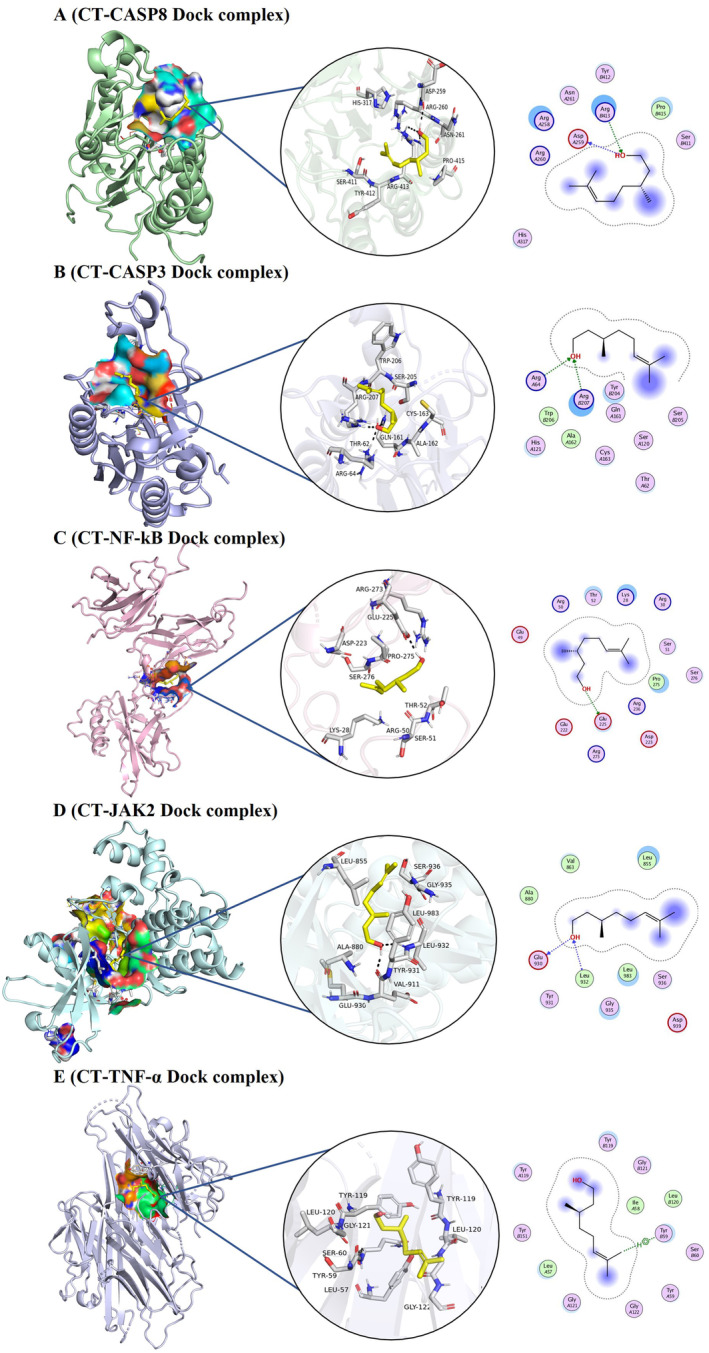
Representation of molecular docking of CT with target proteins. 2D and 3D structures of CT with (A) Caspase‐8 (B) Caspase‐3 (C) NF‐κB (D) JAK2 (E) TNF‐α.

**FIGURE 2:**
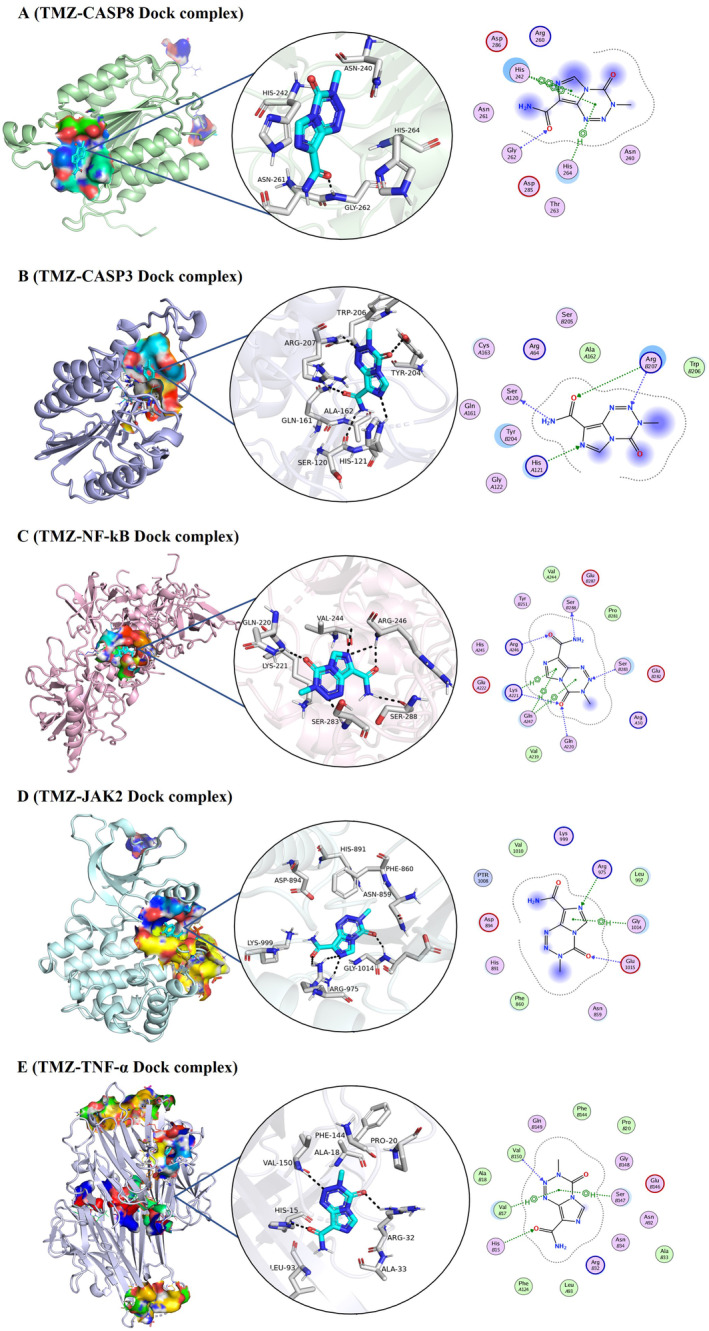
Molecular docking of TMZ with target proteins. 2D and 3D structures of TMZ with (A) Caspase‐8 (B) Caspase‐3 (C) NF‐κB (D) JAK2 (E) TNF‐α.

The docking studies evaluated the interactions of CT with caspase‐8, caspase‐3, NF‐κB, JAK2, and TNF‐α, comparing its binding efficiency with 5‐FU and TMZ. The docking score of CT with caspase‐8 displayed a stronger binding score of −4.82 kcal/mol and an RMSD of 2.85 Å, forming hydrogen bonds with ASP259 and ARG413. TMZ exhibited a slightly more binding score of −4.75 kcal/mol and a better RMSD of 1.46 Å, interacting with residues like GLY262 and HIS242 through hydrogen and π‐interactions. 5‐FU had a weaker binding score of −4.25 kcal/mol and RMSD of 1.29 Å, showing interactions with residues THR337 and LEU401. CT achieved a strong binding score of −5.25 kcal/mol and an excellent RMSD of 0.76 Å, interacting with ASP64 and ARG207 for caspase‐3. The binding score of TMZ was −4.83 kcal/mol, with an RMSD of 1.45 Å, showing key interactions with SER120, ARG207, and HIS121. 5‐FU had a lower binding score of −4.39 kcal/mol and RMSD of 1.27 Å, interacting with multiple residues such as ARG64, HIS121, and GLN161. For NF‐kB, CT exhibited a binding score of −5.08 kcal/mol and RMSD of 1.41 Å, forming hydrogen bonds with GLU225. TMZ displayed the strongest binding score of −5.60 kcal/mol and RMSD of 1.30 Å, with interactions involving SER288 and ARG246. Meanwhile, 5‐FU had a binding score of −4.11 kcal/mol and RMSD of 2.95 Å, forming weaker interactions with residues ARG273 and LYS28. CT demonstrated a binding score of −5.08 kcal/mol and RMSD of 1.96 Å for JAK2, interacting with GLU930 and LEU932. TMZ had a lower binding score of −4.56 kcal/mol and RMSD of 1.58 Å, with key interactions involving ARG975 and GLU1015. 5‐FU showed a binding score of −4.42 kcal/mol and RMSD of 0.73 Å, interacting with residues GLU898 and PHE995. CT displayed a binding score of −5.17 kcal/mol and RMSD of 1.13 Å for TNF‐α, interacting with TYR59. TMZ showed a slightly weaker binding score of −4.60 kcal/mol and RMSD of 1.37 Å, forming hydrogen and π‐interactions with HIS15 and VAL17. 5‐FU had the lowest binding score of −4.15 kcal/mol and RMSD of 1.89 Å, with weaker interactions involving TYR59 and TYR119 (Table 2, Figures 1 and 2). Usually, a more negative binding score indicates a stable ligand–target interaction and a lower RMSD value suggests greater similarity between interacting proteins. Overall, the binding scores consistently indicated stronger binding affinities for CT compared to TMZ, 5‐FU across various targets. Hence, these interactions might be involved in the inhibition of JAK2, NF‐kB, and TNF‐α and activation of caspases.

**TABLE 2:** Molecular docking analysis of CT with different target proteins.

Compounds	S score (kcal/mol)	RMSD (Å)	Atom of compounds	Atom of receptors	Residue of receptor	Type of interaction bond	Distance (Å)	*E* (kcal/mol)
Caspase‐8 (PDB ID: 3KJQ)								
CT	−4.82	2.85	O‐30	O	ASP259	H‐donor	2.88	−1.7
			O‐30	NH1	ARG413	H‐acceptor	3.03	−4.2
			O‐30	NH2	ARG413	H‐acceptor	3.27	−2.3
5‐FU	−4.25	1.29	N‐3	OG1	THR337	H‐donor	3.28	−0.7
			O‐1	OG1	THR337	H‐acceptor	2.83	−2.1
			O‐6	CB	LEU401	H‐acceptor	3.51	−0.6
			6‐ring	6‐ring	PHE399	pi‐pi	3.73	−0.0
TMZ	−4.75	1.46	O‐1	N	GLY 262	H‐acceptor	2.94	−4.4
			6‐ring	CB	HIS 264	pi‐H	4.64	−0.5
			5‐ring	5‐ring	HIS 242	pi‐H	3.46	−0.0
			6‐ring	5‐ring	HIS 242	pi‐H	3.80	−0.0
Caspase‐3 (PDB ID: 1NME)								
CT	−5.25	0.76	O‐30	NH2	ASP64	H‐acceptor	3.01	−4.4
			O‐30	NE	ARG207	H‐acceptor	2.97	−3.5
5‐FU	−4.39	1.27	N‐3	O	SER120	H‐donor	3.40	−1.8
			O‐1	CA	HIS121	H‐acceptor	3.45	−0.9
			O‐6	NE	ARG64	H‐acceptor	3.50	−0.5
			O‐6	NH2	ARG64	H‐acceptor	2.99	−4.6
			O‐6	NH2	GLN161	H‐acceptor	3.14	−1.7
			O‐6	NE	ARG207	H‐acceptor	3.42	−1.3
TMZ	−4.83	1.45	N‐18	O	SER 120	H‐donor	3.08	−0.9
			O‐1	NE	ARG 207	H‐acceptor	3.33	−1.8
			N‐4	ND1	HIS 121	H‐acceptor	3.18	−1.0
			N‐11	N	ARG 207	H‐acceptor	3.33	−0.5
NF‐κB (PDB ID: 1IKN)								
CT	−5.08	1.41	O‐30	OE2	GLU225	H‐donor	3.02	−2.3
5‐FU	−4.11	2.95	O‐1	NH1	ARG273	H‐acceptor	3.16	−1.4
			O‐1	NH1	ARG273	H‐acceptor	2.91	−5.2
			O‐6	NZ	LYS28	H‐acceptor	2.93	−8.3
			6‐ring	CA	THR52	pi‐H	3.85	−0.5
TMZ	−5.60	1.30	N‐18	O	SER 288	H‐donor	3.03	−1.1
			O‐1	N	ARG 246	H‐acceptor	3.04	−3.6
			O‐9	CA	GLN 220	H‐acceptor	3.51	−0.5
			O‐9	N	LYS 221	H‐acceptor	3.25	−1.1
			N‐11	N	SER 283	H‐acceptor	3.12	−1.9
			5‐ring	CD	LYS 221	pi‐H	3.81	−0.6
			5‐ring	CG	GLN 247	\pi‐H	4.10	−0.5
			6‐ring	CG	GLN 247	pi‐H	4.81	−0.5
JAK2 (PDB ID: 3JY9)								
CT	−5.08	1.96	O‐30	O	GLU930	H‐donor	2.86	−0.9
			O‐30	N	LEU932	H‐acceptor	3.06	−1.9
5‐FU	−4.42	0.73	N‐7	OE2	GLU898	H‐donor	2.96	−9.8
			6‐ring	CA	ASP994	pi‐H	3.53	−1.2
			6‐ring	N	PHE995	pi‐H	4.15	−1.4
TMZ	−4.56	1.58	N‐4	NH1	ARG 975	H‐acceptor	3.27	−4.3
			O‐9	N	GLU 1015	H‐acceptor	3.11	−1.0
			5‐ring	N	GLY 1014	pi‐H	3.86	−1.4
TNF‐α (PDB ID: 2AZ5)								
CT	−5.17	1.13	C‐16	6‐ring	TYR59	H‐pi	3.82	−0.5
5‐FU	−4.15	1.89	N‐3	O	LEU120	H‐donor	3.08	−2.2
			6‐ring	CB	TYR59	pi‐H	4.83	−0.7
			6‐ring	CB	TYR119	pi‐H	4.07	−1.0
TMZ	−4.60	1.37	O‐1	NE2	HIS 15	H‐acceptor	3.31	−1.7
			N‐11	N	VAL 150	H‐acceptor	3.35	−1.8
			6‐ring	CB	VAL 17	pi‐H	3.32	−0.5
			6‐ring	CA	SER 147	pi‐H	3.04	−0.6

Abbreviation: RMSD, root mean square deviation.

### 3.3 Target Proteins of CT Linked With GBM

**FIGURE 3:**
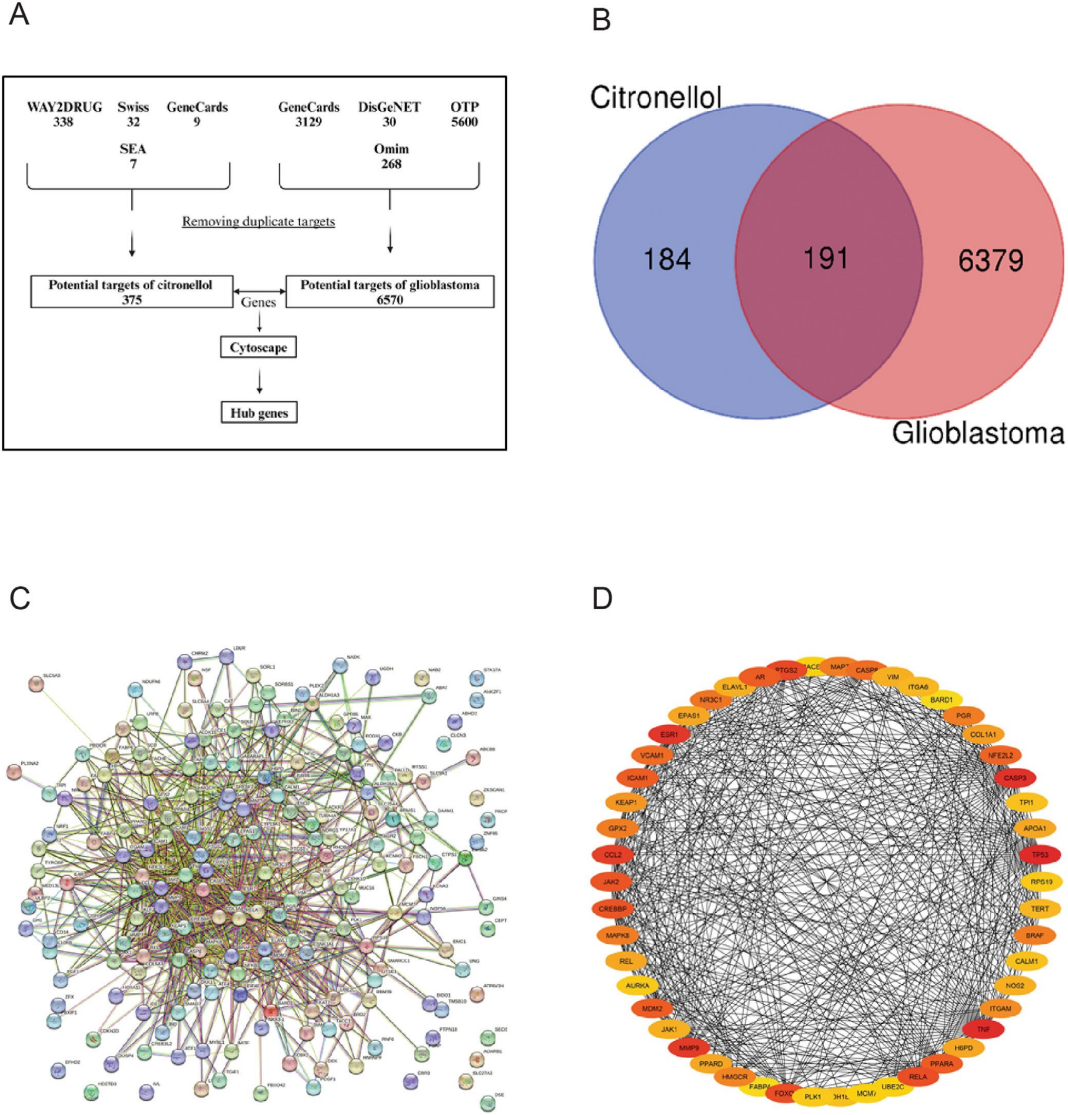
Screening of targets of CT against GBM and PPI network of intersected genes. (A) Flowchart for screening core targets of CT (drug) and GBM (disease) through different databases. (B) Venn diagram: Intersected target genes of CT and GBM. The overlapping region between two circles showed targets that are common to both CT and GBM. (C) PPI network of CT‐GBM intersected genes using STRING database. (D) Topmost 50 molecular targeted based on cytoscape scoring generated by Cytoscape 3.10.2.

CT targets were predicted using a variety of databases, providing 375 possible targets after removing duplication. This is a strong indication that many targets may induce a synergistic effect in the treatment of GBM. A total of 6570 targets were identified for GBM after removing duplication from different public databases. The Venn diagram revealed 191 overlapping targets that were selected between 6570 targets associated with GBM and 375 targets associated with CT. From STRING analysis, 188 unique targets out of 191 overlapping targets were directly related to GBM occurrence and development, indicating 188 nodes and 1016 edges. Cytoscape 3.10.2 was then used to conduct PPI network analysis on the STRING network, revealing a network of interactions. The top 10 targets of CT against GBM included tumor protein 53 (TP53), TNF, caspase‐3, estrogen receptor 1(ESR1), Matrix metalloproteinase‐9 (MMP‐9), C‐C motif chemokine ligand 2 (CCL2), prostaglandin‐endoperoxide synthase 2 (PTGS2), reticuloendotheliosis viral oncogene homolog A (RELA), Forkhead box protein O1 (FOXO1), and JAK2 (Figure 3).

### 3.4 PPI Network Construction and GO Analysis

**FIGURE 4:**
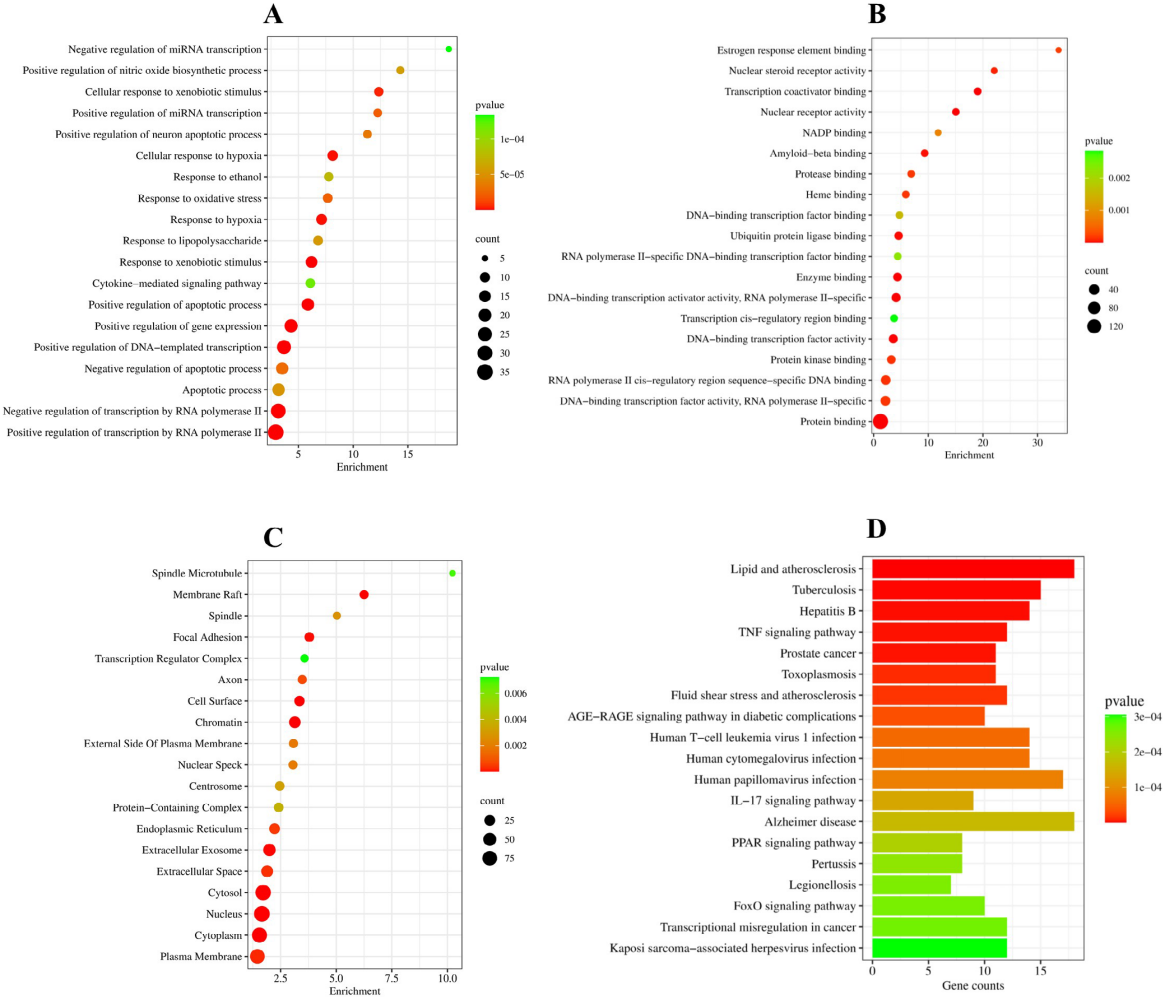
Functional annotation and potential pathways of CT for the treatment of GBM in bubble graph and bar graph generated by SRplot (A) Biological processes. (B) Molecular functions. (C) Cellular components. The number of genes enriched in each pathway is indicated by the size of each bubble. A larger bubble indicates a higher number of genes implicated in the pathway. (D) KEGG pathway: The top 20 items of KEGG pathway. Color of each bar represents the adjusted *p*‐value of each GO term.

The PPI network connected 188 GBM‐related targets depending on their degree and pathways. GO function analysis identified the biological process (BP), cellular composition (CC), and molecular function (MF) entries for hub genes. Topmost 20 entries of biological process included response to estradiol, positive regulation of apoptotic process, positive regulation of transcription by RNA polymerase II, positive regulation of DNA‐templated transcription, positive regulation of gene expression, negative regulation of transcription by RNA polymerase II, response to xenobiotic stimulus, response to hypoxia, cellular response to hypoxia, cellular response to xenobiotic stimulus, positive regulation of miRNA transcription, response to oxidative stress, negative regulation of apoptotic process, positive regulation of neuron apoptotic process, apoptotic process, response to lipopolysaccharide, positive regulation of nitric oxide biosynthetic process, response to ethanol, cytokine‐mediated signaling pathway, and negative regulation of miRNA transcription. Topmost 20 entries of molecular function included identical protein binding, transcription coactivator binding, protein binding, DNA‐binding transcription activator activity, RNA polymerase II‐specific, nuclear receptor activity, enzyme binding, DNA‐binding transcription factor activity, ubiquitin protein ligase binding, amyloid‐beta binding, nuclear steroid receptor activity, RNA polymerase II cis‐regulatory region sequence‐specific DNA binding, protein kinase binding, DNA‐binding transcription factor activity, RNA polymerase II‐specific, heme binding, protease binding, estrogen response element binding, NADP binding, DNA‐binding transcription factor binding, RNA polymerase II‐specific, DNA‐binding transcription factor binding, and transcription cis‐regulatory region binding. Topmost 20 entries of cellular components included nucleoplasm, chromatin, cytosol, nucleus, membrane raft, cytoplasm, cell surface, extracellular exosome, focal adhesion, plasma membrane, extracellular space, endoplasmic reticulum, axon, external side of plasma membrane, nuclear speck, spindle, centrosome, protein‐containing complex, spindle microtubule, and transcription regulator complex. A KEGG pathway enrichment analysis identified 90 linked signaling pathways. Some of these include cancer pathways, lipid‐metabolism related pathways such as lipid and atherosclerosis, tuberculosis disease, hepatitis B disease, TNF signaling pathway, prostate cancer disease, toxoplasmosis disease, fluid shear stress and atherosclerosis, AGE‐RAGE signaling pathway in diabetic complications, and human T‐cell leukemia virus 1 infection disease (Figure 4).

These results firmly imply that CT differentially regulate biomarkers of apoptosis, thereby enhancing the molecular basis of its therapeutic actions.

### 3.5 CT Exhibited Cytotoxicity in a Dose‐Dependent Manner

**FIGURE 5:**
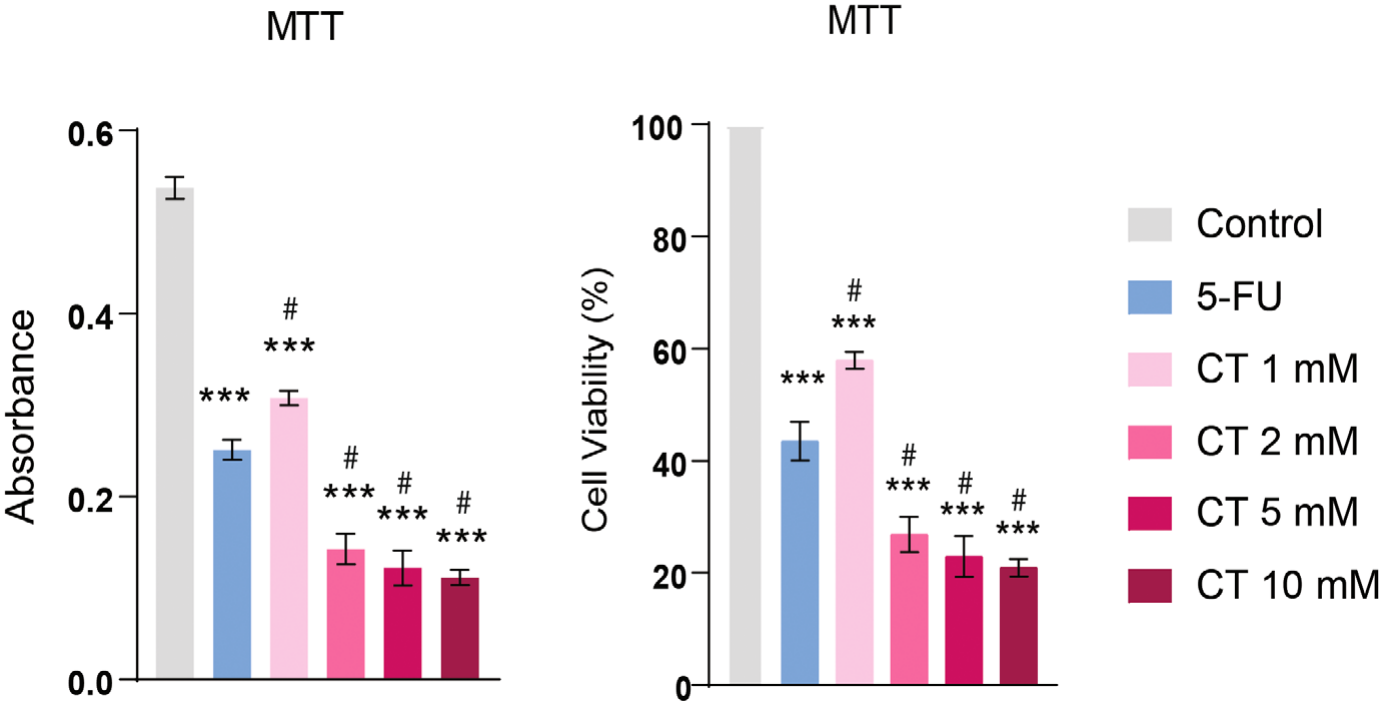
Effect of CT on cell viability. MTT assay showed that CT has a significant effect on cell viability at the indicated doses (1, 2, 5, and 10 mM). At doses above 1 mM, a greater reduction in cell viability was observed. One‐way ANOVA followed by Tukey's multiple comparison test, *n* = 3, ***≤ 0.001 (treated groups vs. control); #≤ 0.001 (CT‐treated groups vs. 5‐FU‐treated group).

Initially, an MTT assay was performed to identify the cytotoxic dose of CT in human SF767 cells. SF767 cells were treated with ascending concentrations of CT (1, 2, 5, and 10 mM) for 24 h and our results revealed that CT exhibited prominent cytotoxicity above 1 mM and decreased cell viability in a dose‐dependent fashion with an IC50 value of 1.3 mM. IC50 measurements are used to calculate the dose of a chemotherapeutic agent required to kill 50% of the cell population. A reduction in cellular viability is indicative of increased cytotoxicity and vice versa. At doses of 2–10 mM, CT‐treated groups showed more cytotoxicity (*p* < 0.001) as compared to 5‐FU‐treated group (Figure 5 and Figure S3). CV assay was also used to analyze the cytotoxic effects of CT. CT at a dose of 2 mM exhibited significant cytotoxic effects which were comparable to 5‐FU‐treated group. Moreover, at doses of 5 and 10 mM, a significant reduction in cellular viability (*p* < 0.001) was observed in CT‐treated groups, which indicated a prominent cytotoxic effect and this effect was much more pronounced than in the 5‐FU‐treated group (Figure S4).

### 3.6 CT Induced Cell Death in SF767 Cell Line

**FIGURE 6:**
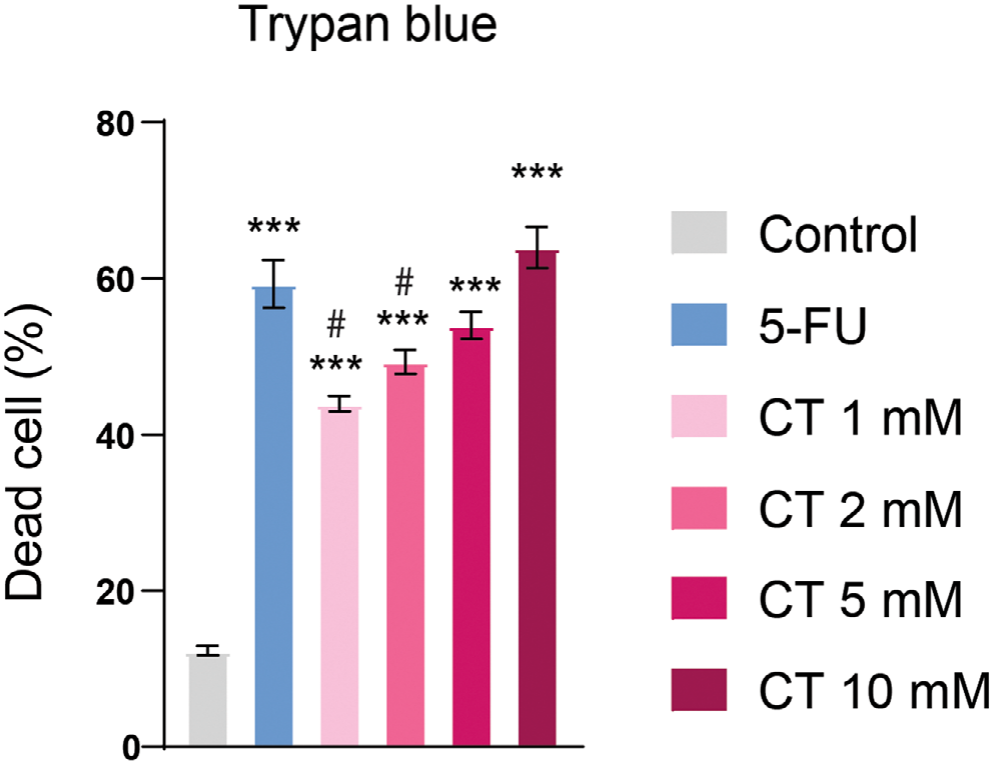
CT induced cell death. Trypan blue staining showed an increased cell death with CT treatment. One‐way ANOVA followed by Tukey's multiple comparison test, *n* = 3, ***≤ 0.001 (treated groups vs. control); #≤ 0.001 (CT‐treated groups vs. 5‐FU‐treated group).

The Trypan blue assay revealed that over 50% of SF767 cells experienced cell death due to CT treatment. CT treatment dose dependently induced cell death and at a dose of 5 and 10 mM, CT effect was comparable to 5‐FU. However, at lower doses (1 and 2 mM), CT‐treated groups displayed less cell death as compared to 5‐FU (Figure 6).

### 3.7 CT Upregulated the Levels of Antioxidants

**FIGURE 7:**
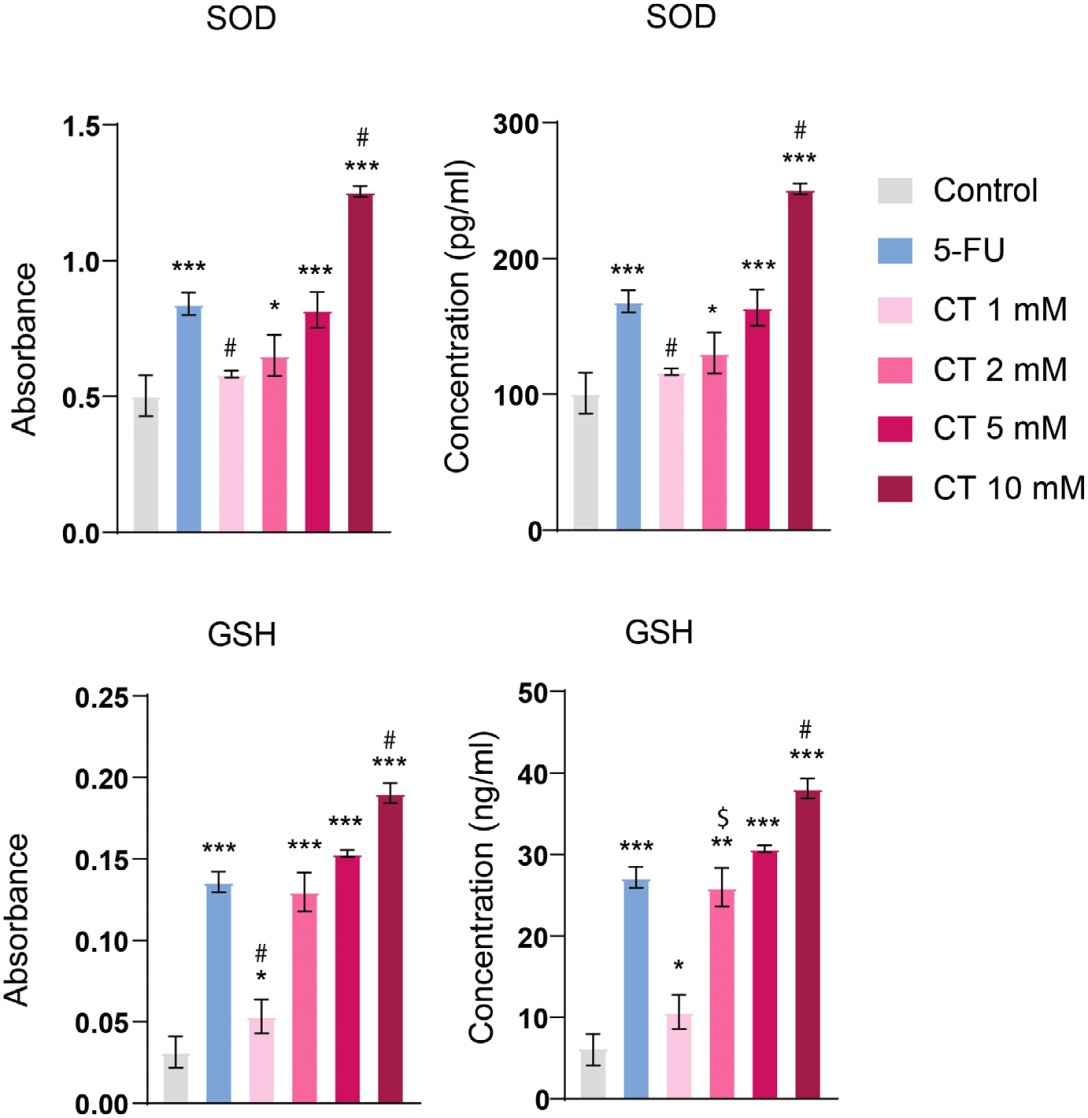
CT dose dependently increased GSH and SOD levels. One‐way ANOVA followed by Tukey's multiple comparison test, *n* = 3, ***≤ 0.001, **≤ 0.01, *≤ 0.05 (treated groups vs. control); #≤ 0.001, $≤ 0.01 (CT‐treated groups vs. 5‐FU‐treated group).

Apoptosis is known to reduce inflammation and oxidative stress. We, therefore, assessed the effect of CT on the levels of antioxidants (GSH and SOD). The findings demonstrated that CT at higher doses prominently induced GSH and SOD levels and at a dose of 10 mM, CT showed superior effects (*p* < 0.001) as compared to 5‐FU. GSH and SOD are well‐known antioxidants that scavenge free radicals and thereby attenuate oxidative stress. These findings indicate that CT reduced oxidative stress in a dose‐dependent fashion by raising the levels of GSH and SOD (Figure 7).

### 3.8 CT Enhanced Caspase‐3 and Annexin‐V Levels

**FIGURE 8:**
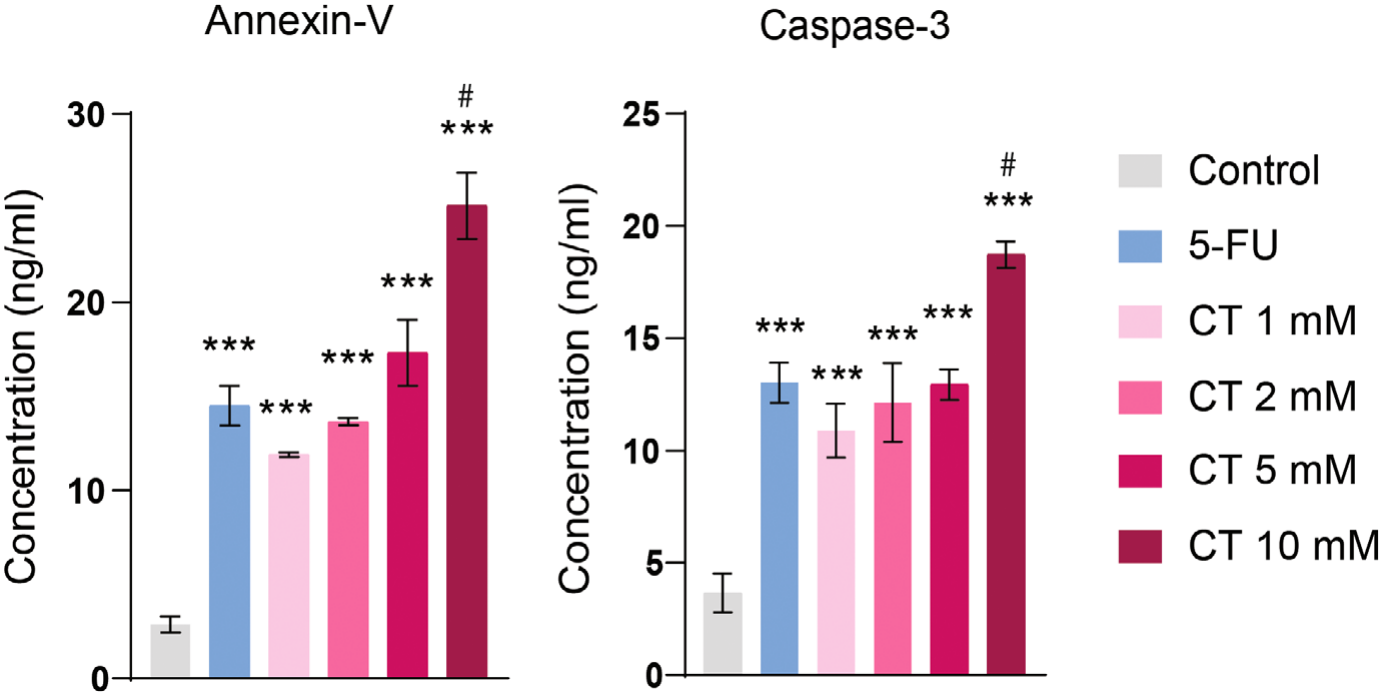
Representation of apoptotic effect of CT. CT treatment enhanced caspase‐3 and annexin‐V levels in a dose‐dependent manner. One‐way ANOVA followed by Tukey's multiple comparison test, *n* = 3, ***≤ 0.001, **≤ 0.01 (treated groups vs. control); #≤ 0.001 (CT‐treated groups vs. 5‐FU‐treated group).

The application of CT and 5‐FU resulted in a notable upregulation of caspase‐3 and annexin‐V levels in SF767 cells, relative to the untreated control group. Caspase‐3 and annexin‐V are established biomarkers of apoptosis and therefore, the current observation serves as a compelling evidence for the induction of apoptosis by CT. Furthermore, a dose‐dependent response was discerned, with higher concentrations of CT exhibiting a significantly heightened and more efficacious impact compared to equimolar doses of 5‐FU. CT displayed superior effects (*p* < 0.001) at a dose of 10 mM when compared to 5‐FU‐treated group (Figure 8).

### 3.9 CT Induced Transcript Levels of Apoptotic Genes and Attenuated Proinflammatory Genes

**FIGURE 9:**
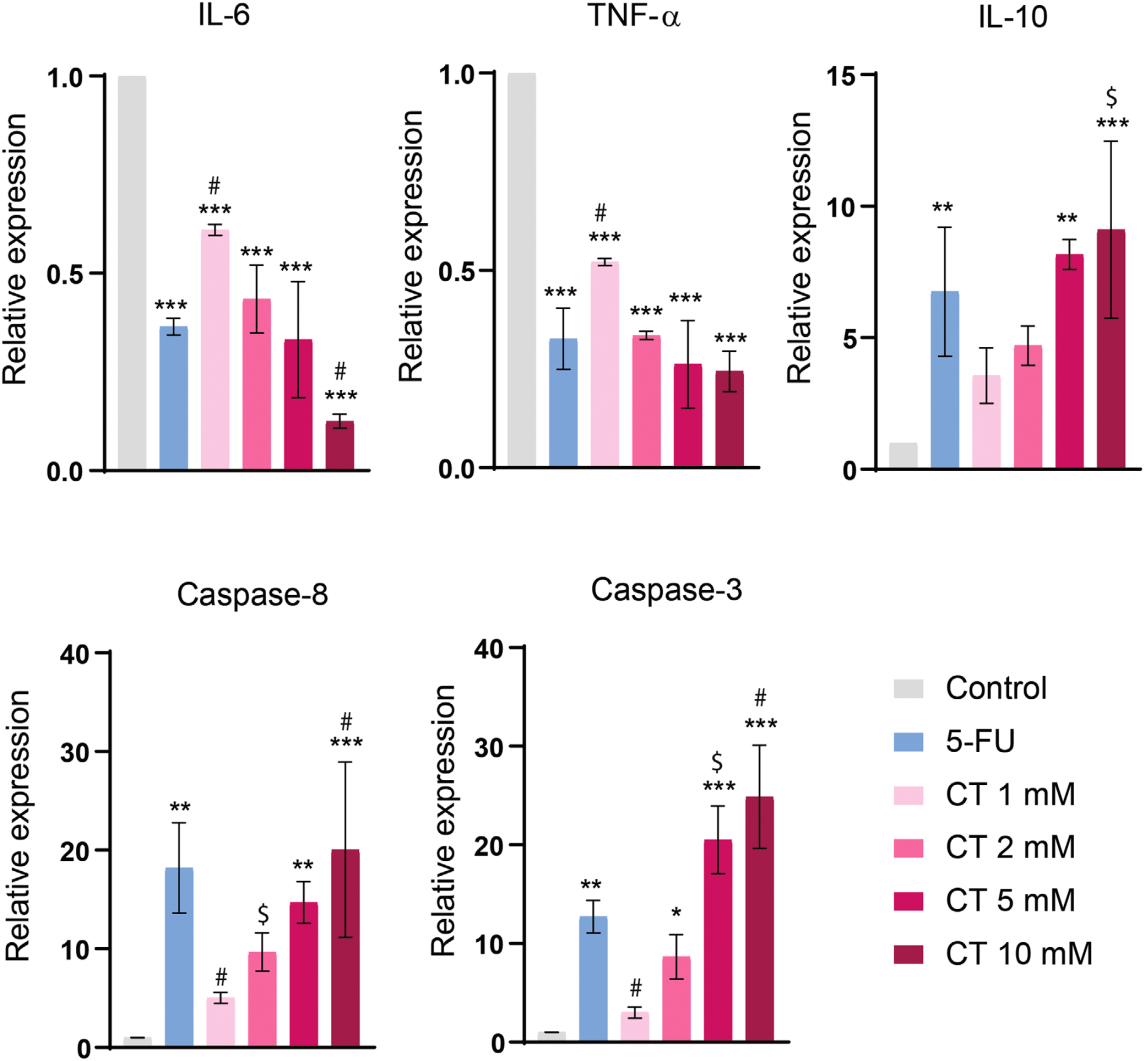
CT demonstrated anticancer effects via elevation of apoptotic genes. SF767 cells were treated with CT for 24 h. RT‐PCR was carried out to examine relative expression by the ΔΔCT method. A prominent upregulation in IL‐10, caspase‐3, and caspase‐8 while a downregulation in IL‐6 and TNF‐α was observed in cells treated with CT and 5‐FU. One‐way ANOVA followed by Tukey's multiple comparison tests, *n* = 3, ***≤ 0.001, **≤ 0.01, *≤ 0.05 (treated groups vs. control); #≤ 0.001, $≤ 0.01 (CT‐treated groups vs. 5‐FU‐treated group).

To investigate the molecular mechanism of CT's anticancer effect, the relative mRNA expressions of biomarkers such as interleukin (IL)‐6, IL‐10, caspase‐3, caspase‐8, and tumor necrosis factor‐alpha (TNF‐α) were evaluated. We observed a significant dose‐dependent reduction in transcript levels of proinflammatory biomarkers (IL‐6 and TNF‐α) upon treatment with CT. CT's anti‐inflammatory effects were comparable to 5‐FU‐treated group at doses of 2 and 5 mM and at a dose of 10 mM, its effects were superior (*p* < 0.001) to 5‐FU‐treated group. IL‐10 is an anti‐inflammatory cytokine and CT at higher doses (5 and 10 mM) significantly raised its transcript levels when compared to normal control. Moreover, at a dose of 10 mM, CT demonstrated more pronounced effect (*p* < 0.001) when compared to 5‐FU‐treated group. Similarly, CT treatment dose dependently increased the levels of proapoptotic genes (caspase‐3 and caspase‐8) and at a dose of 10 mM, a much more pronounced effect was witnessed when compared to 5‐FU‐treated group. These findings reflect the anticancer potential of CT owing to its modulatory effects on apoptotic and inflammatory genes (Figure 9).

## 4 Discussion

GBM is one of the highly aggressive and malignant cancers of CNS. The most prevalent cell type in the CNS “glial cells” are the source of GBM tumors (Urbańska et al. 2014). Current researches report that genetic mutations that accumulate over time enable cells to evade immune system destruction which leads to the development of brain tumors. Any agents—chemical, physical, or biological that damage DNA are potential neurocarcinogens, even if these changes may be partially or completely inherited. Aside from genetic predisposition, exposure to elevated levels of ionizing radiation is the most well‐established environmental risk factor for brain tumors (Pagar and Mahale 2023).

Radiation and chemotherapy are commonly employed following surgery, if it is possible, to treat GBM. Other experimental treatments include gene/antibody therapy, the use of angiogenesis inhibitors in conjunction with chemotherapy, and both passive and active immunotherapy. But since the blood–brain barrier makes it difficult to enter the brain, none of the above‐mentioned treatments have been successful in treating this disease (Jovčevska, Kočevar, and Komel 2013).

Previous studies have proven that CT has potent anti‐inflammatory and antioxidant effects in rodents, which also account for its inhibitory impact on *Candida albicans* , *Salmonella typhi*, and *Staphylococcus aureus* . Research indicates that administering CT to patients undergoing chemotherapy and/or radiation therapy mitigates the negative effects of treatment, including nausea, dyspepsia, numbness in the extremities, and hearing impairment. Additionally, it attenuates the reduction of leukocytes and neutrophils, so enhancing their immune response (Yu et al. 2019).

We investigated the anticancer effects of CT in GBM cell line based on its current in silico findings. MTT assay was employed to evaluate cell viability and proliferation. It is the standardized procedure which has been developed for adherent or nonadherent cell cultured in several wells (Kumar, Nagarajan, and Uchil 2018). In our findings, this assay allowed us to determine its effects on GBM cell growth. By exposing cancer cells to different concentrations (1, 2, 5, and 10 mM) of CT and measuring the reduction of MTT into a colored formazan product, we assessed the viability of cancer cells. A decrease in cell viability indicated a potential antiproliferative effect of CT, which is crucial in evaluating its suitability as an anticancer agent. IC50 measurements are used to determine the dose of a chemotherapeutic agent needed to kill 50% of a cell population starting from a specific initial cell count. This concept is understood in MTT analog assays as the dose necessary to inhibit (or kill) 50% of a cell population with an unknown or fluctuating initial cell number. Low IC50 value indicates more potent effect (He et al. 2016). In our findings, CT showed prominent reduction in cellular proliferation with an IC50 value of 1.3 mM indicating its high potency.

Another rapid and adaptive method for determining the cell viability in a range of stimulation scenario is CV staining. The assay may include chemical inhibitors of caspases and/or necroptosis. Alternatively, to address the nature of cell death more precisely, molecular studies (such as overexpression or knockdown) can be carried out (Feoktistova, Geserick, and Leverkus 2016). The assay can provide insights into CT's impact on cell attachment and growth characteristics. In our study, a decrease in CV staining indicated impaired cell adhesion and proliferation.

In this research, we used the ELISA method to measure the levels of annexin‐V and caspase‐3. An antibody's specific interaction with its associated antigen is the basis for the analytical procedures, especially ELISA (Zhou et al. 2022). Annexins are proteins that bind phospholipids and are controlled by calcium (Ca^2+^). They play a major role in several physiological and pathological cellular processes, including inflammation, signal transduction, apoptosis, and autophagy, in addition to membrane‐related activities like exocytosis (Xu et al. 2022). We observed an increase in annexin‐V levels in SF‐767 cells post‐CT treatment indicating the activation of apoptotic processes. This activation is likely associated with the involvement of caspase‐3, a critical executioner caspase known for its pivotal role in mediating and executing the final stages of apoptosis (Jiang et al. 2020). Caspase‐3 and caspase‐8 are important modulators of the programmed cell death response (Pu et al. 2017). CT has shown proapoptotic effects in human mammary tumor cell line (MCF and MDA‐MB‐231) by activating caspase‐9 and caspase‐7 (Rajendran, Pachaiappan, and Thangarasu 2021). Our qPCR result also exhibited increased levels of caspase‐3 and caspase‐8 in CT‐treated SF767 cells which indicated that its proapoptotic effect might be mediated by upregulation of these mediators.

In addition, an important part of the pathophysiology of GBM is the aberrant activation of JAK2 mediated phosphoinositide 3‐kinase (PI3K) pathway. The PI3K antagonist (PTEN), which is inactivated by loss of heterozygosity in 70% and mutation in 40% of primary GBMs, can be targeted at the level of receptor tyrosine kinase activation and downstream PI3K signaling, including AKT amplification (Stegh et al. 2008). Our in silico findings also reported JAK2 inhibitory potential of CT which might be responsible for its apoptotic effects.

Activated immune cells, specifically monocytes/macrophages and T‐cell subsets, release IL‐10, an essential cytokine that reduces inflammation. The most significant benefit of IL‐10 is its ability to reduce inflammation. By inhibiting immune cell activation and reducing the synthesis of inflammatory chemicals, it helps in the regulation of the immune response. The protective role of IL‐10 against inflammation has significance in preventing tissue damage and excessive responses by the immune system (Munir et al. 2023). Our study also showed an upregulation of IL‐10 in CT's treated cells, which is in line with the previous data that reports induction of immune response by CT. IL‐6 is one of the significant cytokines present in the tumor microenvironment at high concentrations and is known to be upregulated in cancer. The strong correlation between inflammation and cancer is demonstrated by the elevated levels of IL‐6 in the tumor microenvironment. IL‐6 stimulates the growth of tumors by controlling various signaling pathways and cancer hallmarks, such as angiogenesis, invasiveness and metastasis, survival, proliferation, apoptosis, and especially, metabolism (Kumari et al. 2016). Our studies also showed overexpression of IL‐6 gene in untreated cancer cells and CT efficiently reduced its expression indicating its beneficial effects in the treatment of GBM.

A common inflammatory cytokine found in the tumor microenvironment is TNF‐α. Tumor‐associated macrophages are the main source of TNF‐α secretion, which initiates chronic inflammation (Zhao and Zhang 2018). Global databases revealed that TNF‐α was highly expressed in a variety of tumor types, including GBM. Numerous studies indicate that TNF‐α and NF‐κB p65 (RelA) are essential for in vitro GBM invasion and infiltration capabilities (Ahsan et al. 2023). Our study also showed a downregulation in the levels of NF‐κB and TNF‐α upon CT treatment, which thereby, supported the previous findings. Moreover, the reduced levels of IL‐6 and TNF‐α also indicate that CT treatment might be beneficial in controlling the invasiveness or proliferation of GBM.

**FIGURE 10:**
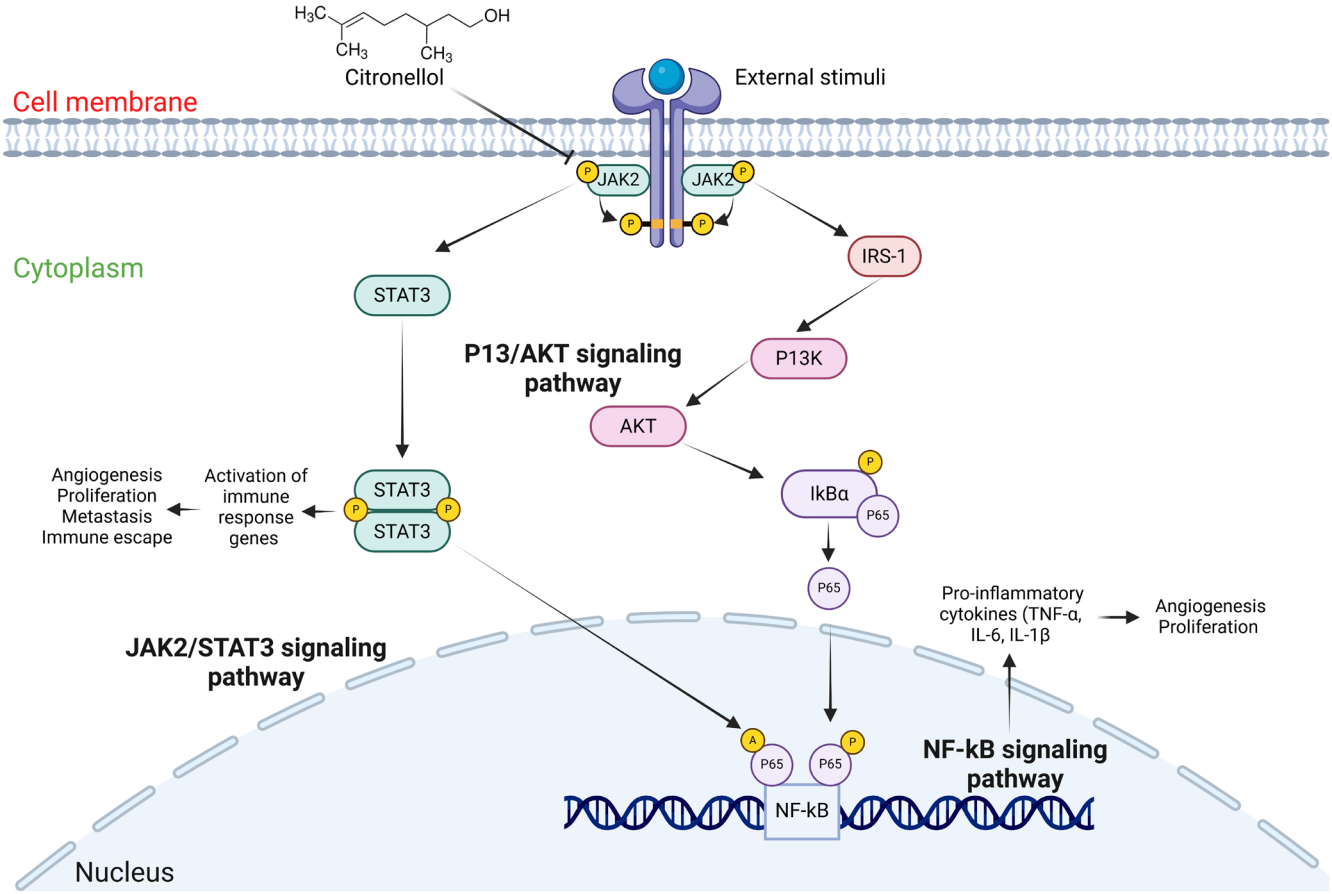
CT regulating cross‐talk between JAK2 and NF‐κB.

Additionally, the above discussion also suggests that CT regulates the cross‐talk between JAK2 and NF‐κB. JAK2 is known to phosphorylate NF‐κB p65 (RelA) via AKT pathway and induce NF‐κB‐mediated transcription of pro‐survival, angiogenic, and inflammatory genes (Chen et al. 2023). Moreover, studies have shown that JAK2 activation leads to phosphorylation of signal transducer and activator of transcription (STAT)3 and phosphorylated STAT3 can acetylate NF‐κB p65 (RelA) and induce NF‐κB‐facilitated transcription (Fan, Mao, and Yang 2013; Lee et al. 2009). Our in silico findings have demonstrated that CT can inhibit JAK2 which will lead to inhibition of JAK/STAT, AKT, and NF‐κB pathways, ultimately resulting in anticancer and anti‐inflammatory effects (Figure 10).

By employing a combination of in silico approaches, cell viability assays, and molecular studies, we have assessed not only the anticancer effect of CT but also its impact on cellular processes, molecular pathways, and gene expression patterns within cancer cells. These findings will contribute to the growing body of knowledge surrounding CT as a potential anticancer agent.

## 5 Conclusion

Based on the above data, it can be concluded that CT possesses strong anticancer effects which may be linked to the upregulation of proapoptotic mediators (caspase‐3 and caspase‐8) and downregulation of inflammatory and pro‐survival modulators (JAK2, NF‐κB). However additional research is necessary to evaluate the precise mechanism of action, pharmacokinetics, and pharmacodynamics profiles of CT in animal models in order to validate the current findings.

## Author Contributions

**Muhammad Nasir Hayat Malik:** conceptualization (equal), data curation (equal), formal analysis (equal), project administration (equal), resources (equal), supervision (equal), writing – review and editing (equal). **Sufyan Ali:** formal analysis (equal), investigation (equal), methodology (equal), software (equal), visualization (equal), writing – original draft (equal). **Amir Ali:** data curation (equal), formal analysis (equal), investigation (equal), methodology (equal), software (equal), validation (equal), visualization (equal). **Abdullah R. Alanzi:** data curation (equal), formal analysis (equal), funding acquisition (equal), investigation (equal), methodology (equal), software (equal), validation (equal), writing – original draft (equal). **Muhammad Atif:** data curation (equal), formal analysis (equal), investigation (equal), methodology (equal), software (equal), validation (equal). **Hattan A. Alharbi:** data curation (equal), formal analysis (equal), investigation (equal), resources (equal), software (equal), validation (equal), visualization (equal). **Bowen Wang:** data curation (equal), formal analysis (equal), investigation (equal), software (equal), validation (equal), visualization (equal). **Moosa Raza:** data curation (equal), formal analysis (equal), investigation (equal), methodology (equal), software (equal), visualization (equal). **Tahir Maqbool:** conceptualization (equal), formal analysis (equal), investigation (equal), methodology (equal), resources (equal), software (equal), validation (equal), visualization (equal). **Irfan Anjum:** conceptualization (equal), data curation (equal), formal analysis (equal), investigation (equal), software (equal), validation (equal), visualization (equal). **Shah Jahan:** conceptualization (equal), data curation (equal), formal analysis (equal), investigation (equal), methodology (equal), resources (equal), software (equal), validation (equal), visualization (equal). **Saud O. Alshammari:** data curation (equal), formal analysis (equal), investigation (equal), resources (equal), software (equal), validation (equal), visualization (equal), writing – review and editing (equal). **Gideon F. B. Solre:** conceptualization (equal), data curation (equal), methodology (equal), project administration (equal), resources (equal), software (equal), validation (equal), visualization (equal), writing – review and editing (equal).

## Conflicts of Interest

The authors declare no conflicts of interest.

## Supporting information


Data S1.

## Data Availability

The datasets used and/or analyzed during the current study are available from the corresponding author on reasonable request.
