# Supplementary material for: Citronellol Induces Apoptosis via Differential Regulation of Caspase‐3, NF‐κB, and JAK2 Signaling Pathways in Glioblastoma Cell Line
Source: Food Sci Nutr. 2025 Jan 6;13(1):e4678. doi: 10.1002/fsn3.4678 (PMC11717069; doi:10.1002/fsn3.4678)
Supplement: Supplementary file 1 — Data S1. [file FSN3-13-e4678-s001.docx]

**Citronellol induces apoptosis via differential regulation of caspase-3, NF-κB and JAK2 signaling pathways in glioblastoma cell line**

**Running Title: Citronellol: A promising anti-cancer agent**

Muhammad Nasir Hayat Malik^1#^, Sufyan Ali^1#^, Amir Ali^1#^, Abdullah R. Alanzi^2^, Muhammad Atif^1^, Hattan A. Alharbi^2^, Bowen Wang^3^, Moosa Raza^1^, Tahir Maqbool^4^, Irfan Anjum^5^, Shah Jahan^6^, Saud O. Alshammari^7^, Gideon F. B. Solre^8*^

^1^Faculty of Pharmacy, The University of Lahore, Lahore 54000, Pakistan

^2^Department of Pharmacognosy, College of Pharmacy, King Saud University, Riyadh, Saudi Arabia

^3^College of Chinese Medicine, Hubei University of Chinese Medicine, Hubei, China

^4^Institute of Molecular Biology and Biotechnology (IMBB), The University of Lahore, Lahore 54000, Pakistan

^5^Shifa College of Pharmaceutical Sciences, Shifa Tameer-e-Millat University, Islamabad 44000, Pakistan

^6^Department of Immunology, University of Health Sciences, Lahore 54000, Pakistan

^7^Department of Pharmacognosy and Alternative Medicine, College of Pharmacy, Northern Border University, Rafha, Saudi Arabia

^8^Department of Chemistry, Thomas J. R. Faulkner College of Science and Technology, University of Liberia, Monrovia, Montserrado County, Liberia

^#^These authors contributed equally

^*^Corresponding Author:

Gideon F. B. Solre

Department of Chemistry, Thomas J. R. Faulkner College of Science and Technology, University of Liberia, Monrovia, Montserrado County, Liberia

[gideonfbsolre@gmail.com](mailto:gideonfbsolre@gmail.com); [62834@ul.edu.lr](mailto:62834@ul.edu.lr)

ORCID-ID: 0000-0002-2861-0674


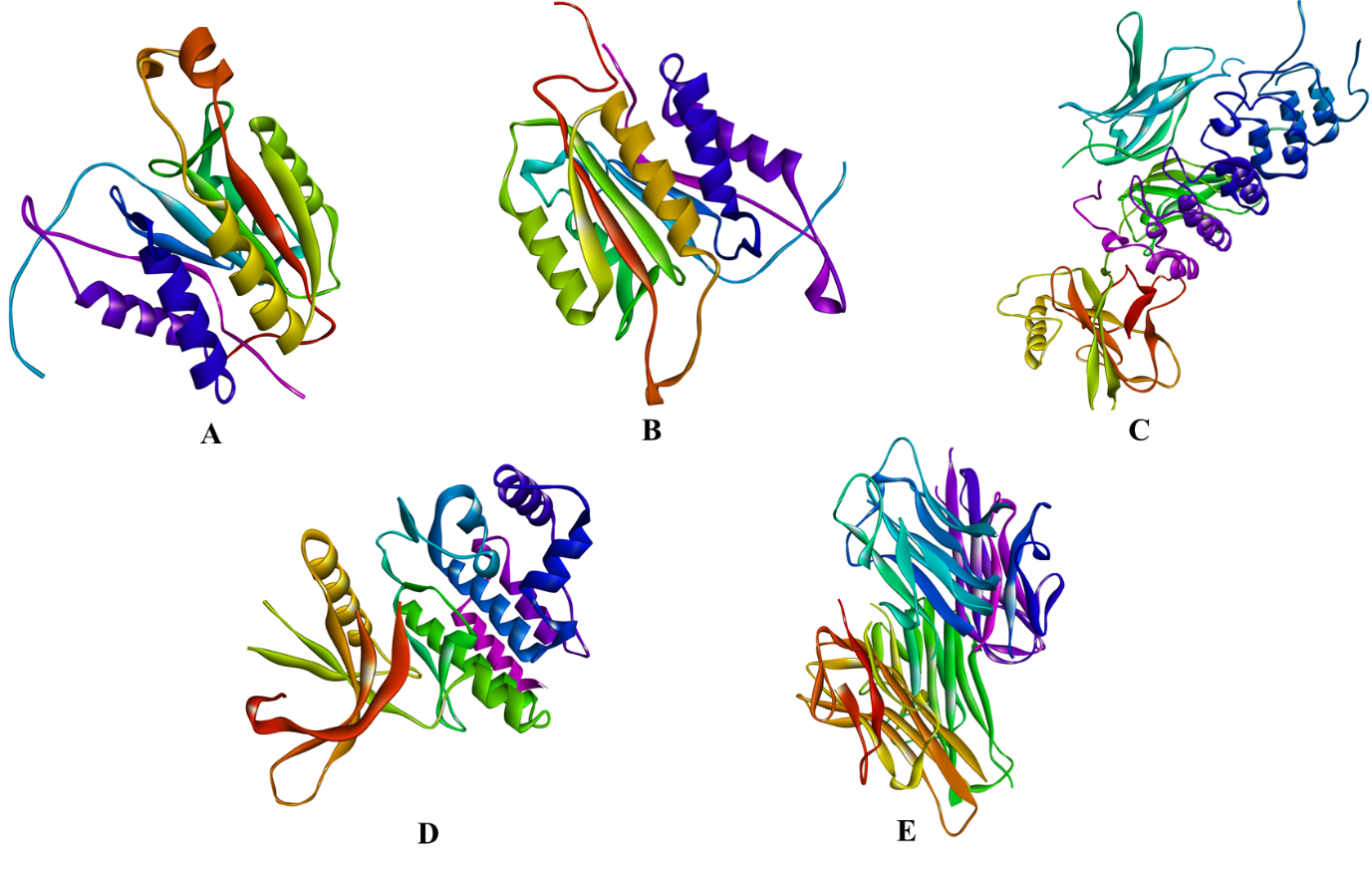


**Figure S1:** **Representation of 3D crystal structures of proteins by Discovery studio**. A) Human caspase-8 B) Human caspase-3 C) Human NF-κB, D) Human JAK2 E) Human TNF-α


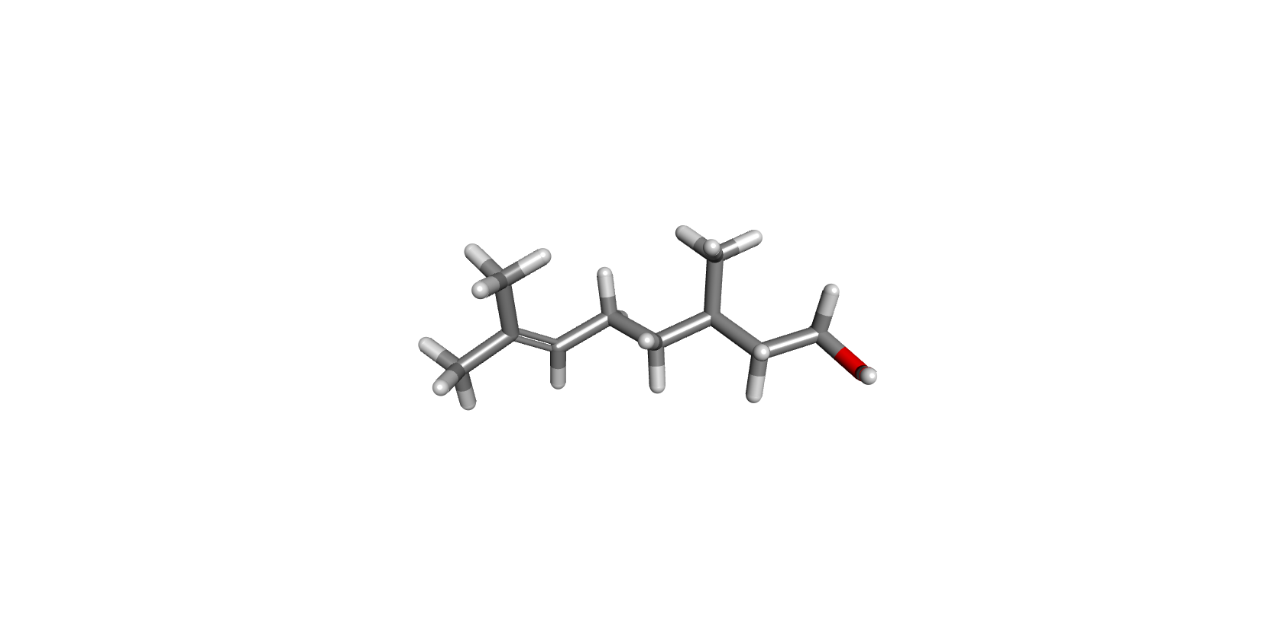


**Figure S2:** **3D structure of ligand (CT)**


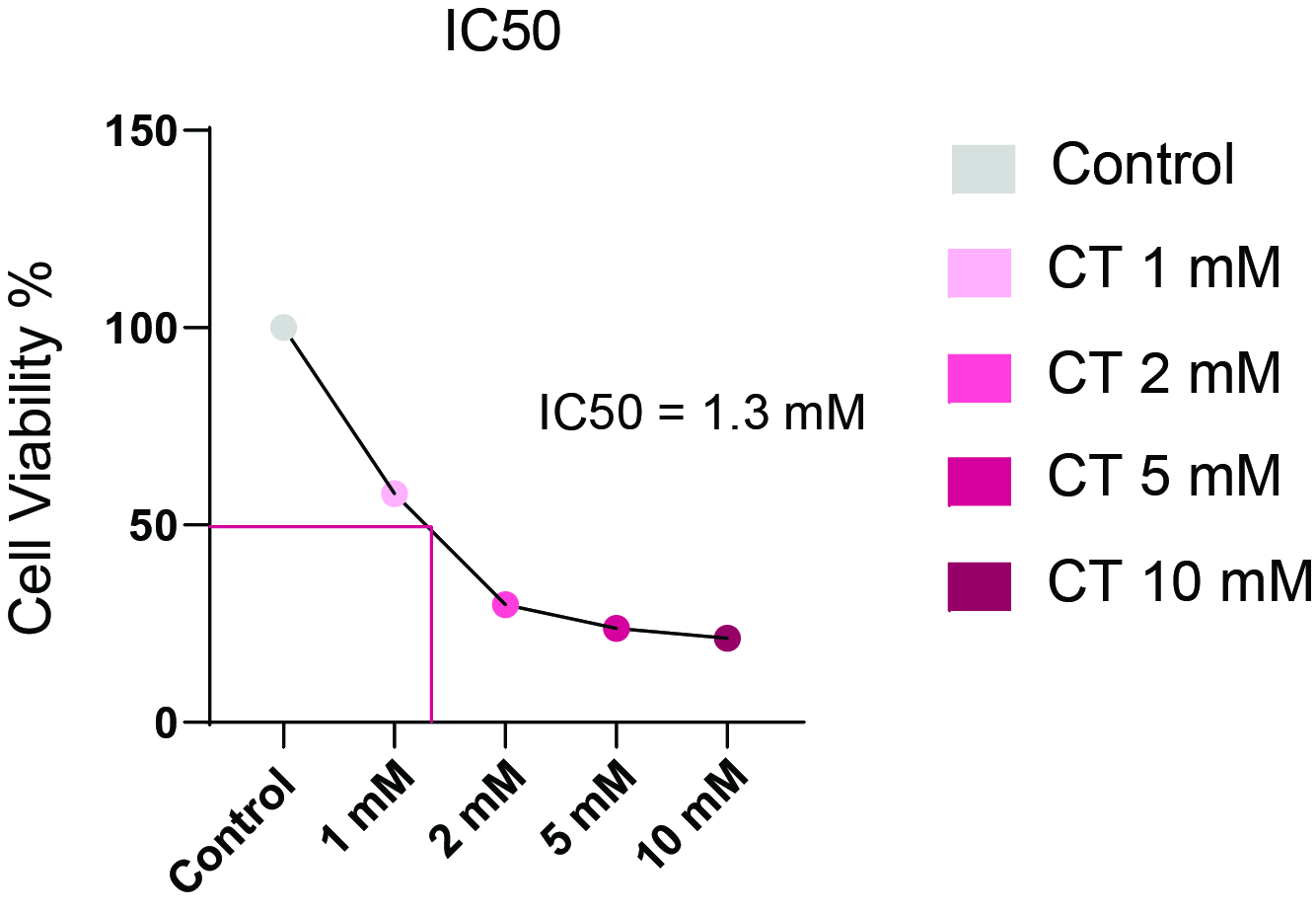


**Figure S3: Graphical representation of IC50 values.** Graphical representation of IC50 values of various concentrations (1 mM, 2 mM, 5 mM, 10 mM) of CT used for assessing viability of SF767 cells. CT demonstrated a strong anti-proliferative effect with the IC50 value of 1.3 mM**.**


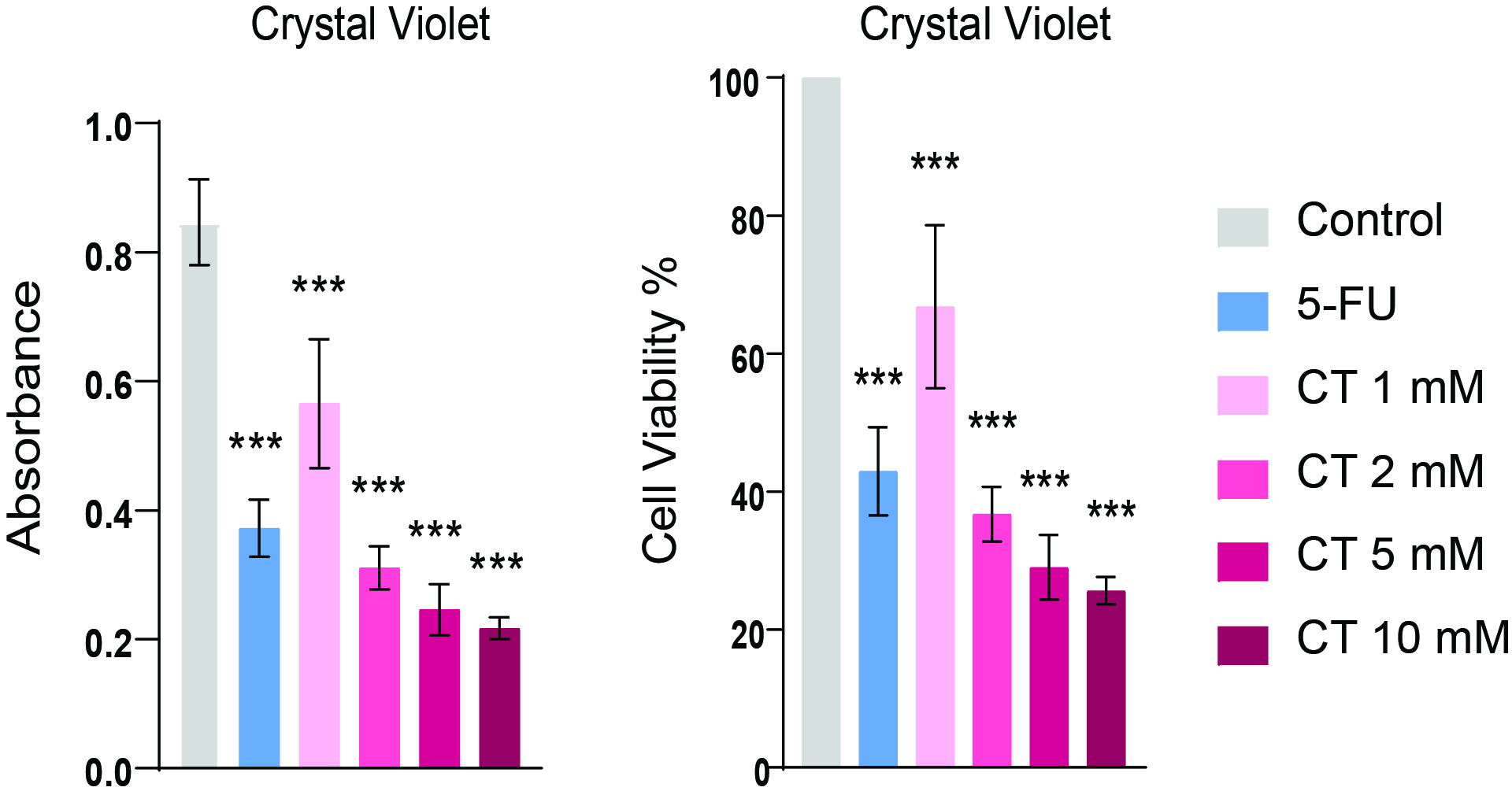


**Figure S4: CV assay displaying the degree of cell viability in various treated and untreated groups.** In comparison to untreated groups, 5-FU and CT reduced cell viability, which demonstrated that CT possesses anti-proliferative activity. One-way ANOVA followed by Tukey ̓s multiple comparison test, n = 3, *** ≤ 0.001.

**Table S1: Binding sites prediction of target proteins**

| **Protein PDB ID** | **Surface area (Å^2^)** | **Volume (Å^3^)** |
| --- | --- | --- |
| 3KJQ (Caspase-8) | 383.489 | 407.435 |
| 1NME (Caspase-3) | 93.543 | 154.001 |
| 1IKN (IKB-α/NF-KB1 complex) | 1133.582 | 943.278 |
| 3JY9 (JAK2) | 949.120 | 755.967 |
| 2AZ5 (TNF-α) | 2743.256 | 4514.068 |
